# A Longitudinal Study of *Escherichia coli* Clinical Isolates from the Tracheal Aspirates of a Paediatric Patient—Strain Type Similar to Pandemic ST131

**DOI:** 10.3390/microorganisms12101990

**Published:** 2024-09-30

**Authors:** Brankica Filipic, Milan Kojic, Zorica Vasiljevic, Aleksandar Sovtic, Ivica Dimkic, Emily Wood, Alfonso Esposito

**Affiliations:** 1 Department of Microbiology and Immunology, Faculty of Pharmacy, University of Belgrade, Vojvode Stepe 450, 11221 Belgrade, Serbia; 2 Institute of Molecular Genetics and Genetic Engineering, University of Belgrade, Vojvode Stepe 444a, 11042 Belgrade, Serbia; mkojic@imgge.bg.ac.rs; 3 Institute of Virology, Vaccines and Sera “Torlak”, Vojvode Stepe 448, 11042 Belgrade, Serbia; 4 Mother and Child Health Institute of Serbia, Faculty of Medicine, University of Belgrade, Radoja Dakića 8, 11070 Belgrade, Serbia; zorica.vasiljevic@gmail.com (Z.V.); adsovtic@gmail.com (A.S.); 5 Department of Biochemistry and Molecular Biology, Faculty of Biology, University of Belgrade, Studentski trg 16, 11158 Belgrade, Serbia; ivicad@bio.bg.ac.rs; 6 Biosciences, College of Life and Environmental Sciences, University of Exeter, Exeter EX4 4QD, UK; e.j.wood@exeter.ac.uk; 7 Department of Medicine and Surgery, “Kore” University of Enna (UKE), Contrada Santa Panasia, 94100 Enna, Italy; alfonso.esposito@unikore.it

**Keywords:** *Escherichia coli*, respiratory tract infections, antimicrobial drug resistance, virulence factors

## Abstract

*Escherichia coli* is a Gram-negative bacterium and part of the intestinal microbiota. However, it can cause various diarrhoeal illnesses, i.e., traveller’s diarrhoea, dysentery, and extraintestinal infections when the bacteria are translocated from the intestine to other organs, such as urinary tract infections, abdominal and pelvic infections, pneumonia, bacteraemia, and meningitis. It is also an important pathogen in intensive care units where cross-infection may cause intrahospital spread with serious consequences. Within this study, four *E. coli* isolates from the tracheal aspirates of a tracheotomised paediatric patient on chronic respiratory support were analysed and compared for antibiotic resistance and virulence potential. Genomes of all four isolates (5381a, 5381b, 5681, 5848) were sequenced using Oxford Nanopore Technology. According to PFGE analysis, the clones of isolates 5681 and 5848 were highly similar, and differ from 5381a and 5381b which were isolated first chronologically. All four *E. coli* isolates belonged to an unknown sequence type, related to the *E. coli* ST131, a pandemic clone that is evolving rapidly with increasing levels of antimicrobial resistance. All four *E. coli* isolates in this study exhibited a multidrug-resistant phenotype as, according to MIC data, they were resistant to ceftriaxone, ciprofloxacin, doxycycline, minocycline, and tetracycline. In addition, principal component analyses revealed that isolates 5681 and 5848, which were recovered later than 5381a and 5381b (two weeks and three weeks, respectively) possessed more complex antibiotic resistance genes and virulence profiles, which is concerning considering the short time period during which the strains were isolated.

## 1. Introduction

*Escherichia coli* strains are associated with intestinal microbiota. However, they can cause intestinal infections as well as extraintestinal infections if they colonise other body sites, resulting in infections such as urinary tract infections, wound infections, pneumonia, cholecystitis, and peritonitis [1].

*E. coli* is not widely recognised as an important pathogen in the respiratory tract, and that is probably why its prevalence as a respiratory pathogen might be underestimated [2,3]. Traditionally, respiratory tract infections have been primarily associated with Gram-positive bacteria; however, the incidence of respiratory infections caused by Gram-negative bacteria is steadily increasing [4]. The common Gram-negative pathogens in lower respiratory tract infections, particularly in the nosocomial setting, are *Pseudomonas aeruginosa*, *Klebsiella pneumoniae*, *Stenotrophomonas maltophilia*, *Acinetobacter baumannii*, and *Achromobacter xylosoxidans*, while the significance of species such as *Chryseobacterium*, *Rhizobium*, *Delftia*, *Elizabethkingia*, *Ralstonia*, *Ochrobactrum*, and several others is poorly defined. These infections are rare in healthy children, but may occur in children with immunodeficiency, cystic fibrosis, primary ciliary dyskinesia, etc. [5]. Moreover, the emergence of multidrug-resistant Gram-negative bacteria represents a major concern, particularly among individuals receiving hospital care. There is an increasing trend of *E. coli* recovery from respiratory tract secretions in recent years and meanwhile some strains of *E. coli* have developed resistance to commonly used antibiotics [6].

The aim of this study was to investigate the antibiotic resistance profiles and virulence potential of four *E. coli* isolates collected from the tracheal aspirate of a 3-year-old boy hospitalised at the Mother and Child Health Care Institute of Serbia “Dr Vukan Čupić”. Although the isolates had been collected in a short three-week period, the goal was to compare them and evaluate the impact of antibiotic therapy on strain adaptation, antibiotic resistance, and virulence phenotype.

## 2. Materials and Methods

### 2.1. Patient and Bacterial Isolates

**Figure 1:**
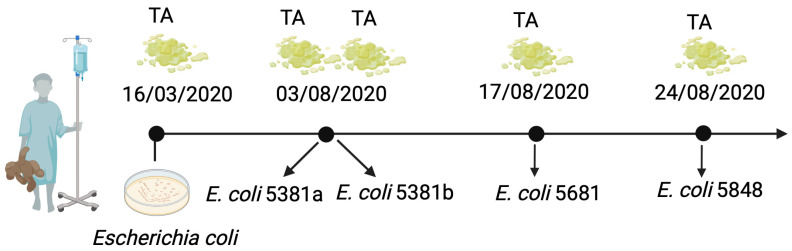
Sample collection and longitudinal bacterial isolation from the hospitalised patient. The first two isolates were not available for this study. TA—Tracheal aspirate. Figure made with Biorender.com, accessed on 27 February 2024.

The patient included in this study was a 3-year-old boy, previously diagnosed with severe birth asphyxia. Since his birth in October 2017, he had been hospitalised at the Mother and Child Health Care Institute of Serbia “Dr. Vukan Čupić”, a University affiliated paediatric tertiary care hospital in Belgrade, Serbia, due to his dependency on mechanical ventilation and other various complications of severe perinatal asphyxia. There were no signs of active lower respiratory tract infection. The clinical course of the patient was complicated by a severe form of hypoxic-ischaemic encephalopathy, seizures, and chronic respiratory failure, requiring prolonged ventilatory support. Over time, the patient was tracheotomised and invasive respiratory support was performed, until his death following a cardiac arrest in December 2021. The patient received gentamicin and cephalexin as prophylaxis and, at one point, as treatment for an intercurrent urinary tract infection. In the course of his hospitalisation, tracheal aspirate surveillance cultures had been sampled periodically and sent for microbiological analysis, which revealed the presence of bacterial species commonly associated with nosocomial colonisation/infection, namely *Acinetobacter baumannii*, *Pseudomonas aeruginosa*, *Proteus mirabilis*, and, later on, *E. coli* in various combinations. These findings were regarded as colonisation since there were no clinical signs of severe lower respiratory infection and the inflammatory parameters were not suggestive of an infection. The first *E. coli* isolate from this patient had been collected in March 2020 and once again in June 2020, but these isolates were not available for the research analysis. However, *E. coli* isolates (*n* = 4) collected during the three week period in August (3 August 2020–24 August 2020) were recovered longitudinally from the tracheal aspirates of the patient and submitted for further analysis (Figure 1). Bacterial identification was performed routinely in the hospital laboratory using standard morphological and biochemical tests (Gram stain, cultivation on different media, etc.) and confirmed as *E. coli* according to genomic data generated in the research laboratory using the Oxford Nanopore sequencing technique. The *E. coli* isolates were cultivated and maintained in Luria-Bertani (LB) medium (Torlak, Belgrade, Serbia), and each isolate was named randomly to preserve anonymity of the patient (isolates: 5381a, 5381b, 5681, 5848) (Table 1). The isolates 5381a and 5381b were recovered on the same day (3 August 2024), while the isolate 5681 was collected 14 days later, and for isolate 5848, 21 days later from the first isolation (Table 1, Figure 1). The study protocol was approved by the Ethics Committee of the Mother and Child Health Care Institute of Serbia “Dr Vukan Čupić”, (approval No. 8/32).

### 2.2. Pulsed Field Gel Electrophoresis (PFGE)

The genetic relatedness among isolates was determined by PFGE analysis of in-gel *Xba*I-digested genomic DNA. DNA preparation and restriction enzyme digestion for PFGE of the *E. coli* isolates was processed as previously described [7]. PFGE was carried out in 0.5 xTBE (45 mmol/L Tris base, 45 mmol/L boric acid, and 1 mmol/L EDTA, pH 8) for 18 h using a contour-clamped homogeneous electric field system (2015 Pulsafor unit; LKB Pharmacia, Bromma, Sweden), as described previously [8].

### 2.3. Oxford Nanopore Genome Sequencing

Total DNA for whole genome sequencing from all of the *E. coli* isolates was obtained using phenol:chloroform extraction. Rapid barcoding of genomic DNA was carried out using the Rapid Barcoding Sequencing Kit (SQK-RBK004) protocol (Oxford Nanopore Technologies-ONT, Oxford, UK), with four replicates of each isolate. The extracted DNA was quantified using a Qubit fluorometer (Thermo Fisher Scientific, Waltham, MA, USA) and loaded into the MinION FLO-MIN106D flow cell, with sequencing performed according to the manufacturer’s instructions. Two separate runs (designated as runs 1 and 2) were performed using the same flow cell. Sequencing data were collected and processed in real time using the MinKNOWTM software version 23.04.5.

De novo genome assembly of the ONT data was performed using Flye, a long-read assembly algorithm [9], and the quality of assembled genomes was checked using Quality Assessment Tool (QUAST version 5.2.0) [10]. Finally, the Racon polishing tool (version 1.4.21, for rapid consensus) was used and enabled the efficient construction of genome sequences with high accuracy [11]. Genomes were deposited in the National Centre for Biotechnology Information (NCBI) under the accession numbers: JBDIFQ010000000 (5381a), JBDIFR010000000 (5381b), JBDIFS010000000 (5681), and JBDIFT010000000 (5848).

### 2.4. Sequence Analyses

To determine the average nucleotide identity (ANI) among the assembled genomes of the four strains, the software pyani (version 0.2.9) was used [12], and the ANI and alignment length were plotted using DiMHepy (https://github.com/lucaTriboli/DiMHepy, accessed on 15 March 2024) [13]. Multi-locus sequence type (MLST) analysis was performed by uploading the assembled genomes onto the webserver of the Centre for Genomic Epidemiology (https://cge.food.dtu.dk/services/MLST/, accessed on 25 March 2024). According to MLST results, the sequences of the four strains were also compared with the reference sequence of the strain NCRC13441 (accession number ERS530440), as it was previously conducted in Decano et al., 2019 [14] and Ludden et al., 2020 [15]. SNPs common to all strains were filtered out. We searched for the mutations occurring in genes involved in clinically relevant phenotypes [16] and plotted the number of mutations for each strain as a heatmap using the R function ‘heatmap’.

In order to compare the *E. coli* genome sequences, Pan-Genome analyses (the comparison of different isolates of the same species) was used, as described previously [17]. A phylogenetic tree was constructed using Roary (version 3.12.0) [18]. Pan-Genome analyses enabled the detection of a number of core genes (hard core genes that were present in more than 99% of tested isolates, and soft core genes that were present in 95–99% of isolates) and accessory genes (shell genes that were present in 15–95% isolates and cloud genes, present in less than 15% of isolates).

Genes that determined the molecular basis of antibiotic resistance were detected by ResFinder tool (version 44.3), that identifies AMR genes, mutations that are associated with AMR, and predicted phenotypes (http://genepi.food.dtu.dk/resfinder, accessed on 8 April 2024) [19].

### 2.5. Principal Component Analysis (PCA)

The screening of virulence factors within the sequenced genomes was performed using the Virulence Factor Database (VFDB) online tool (http://www.mgc.ac.cn/cgi-bin/VFs/v5/main.cgi, accessed on 10 May 2024) [20]. Principal component analysis (PCA) was performed in order to emphasise the variation of detected virulence factors between sequenced *E. coli* genomes, using XLSTAT statistical software, 2023, version 3.3.1 (https://www.xlstat.com/en/, accessed on 10 May 2024).

### 2.6. Antimicrobial Susceptibility Testing

Antimicrobial susceptibility testing was performed following the recommendations of the European Committee on Antimicrobial Susceptibility Testing (EUCAST, version 14.0), or the Clinical and Laboratory Standards Institute (CLSI, 2014) when breakpoints were not available in EUCAST. Minimal inhibitory concentrations (MICs) were determined by microdilution testing in Muller-Hinton broth (Torlak, Belgrade, Serbia) following breakpoints for *Enterobacterales*. Susceptibility was tested against ceftriaxone (0.5–4 μg/mL), meropenem (1–16 μg/mL), ciprofloxacin (0.012–1 μg/mL), amikacin (4–32 μg/mL), gentamicin (1–4 μg/mL), doxycycline (2–32 μg/mL), minocycline (2–32 μg/mL), and tetracycline (2–32 μg/mL). Experiments were performed in triplicate and after 24 h of incubation at 37 °C, cell density was determined by OD_570_ measurements, using a Multiscan FC Microplate Photometer (Thermo Scientific, Waltham, MA, USA) MICs values were appointed to the lowest concentration of tested antibiotic that inhibited bacterial growth.

### 2.7. Biofilm Formation and Swimming Motility Assay

Biofilm formation among the tested isolates was determined using the protocol established by Stepanovic and co-authors (2007) [21] with slight modifications. A 96-well microtitre plate (Tissue Culture Plate, Sarstedt, Nümbrecht, Germany) was filled with 180 μL of LB broth medium and 20 μL of overnight cultures of *E. coli*, adjusted to 0.5 of the McFarland standard (~10^8^ CFU/mL) with a subsequent 1:100 dilution of this suspension, to produce the final tested bacterial inoculum of ~10^6^ CFU/mL. The microtitre plate was incubated for 48 h at 37 °C. After incubation, each well was washed three times with 200 μL of sterile phosphate-buffered saline (PBS; pH 7.2) (Torlak, Belgrade, Serbia) and the remaining bacteria were fixed by drying at 65 °C for 30 min. The biofilms were stained with 0.1% crystal violet (HiMedia Labs Pvt. Ltd., Mumbai, India) for 30 min at room temperature, and the stain was rinsed by washing three times with 1 xPBS then resolubilised with 96% ethanol. The strain *Pseudomonas aeruginosa* PAO1 was used as a positive control as it is a confirmed strong biofilm producer. The quantification of biofilm formation was performed by measuring absorbance at 570 nm using a Multiscan FC Microplate Photometer (Thermo Scientific, Waltham, MA, USA).

The cut-off value (ODc) was established as three SDs above the mean OD of the negative control: ODc = average OD of the negative control + (3 × SD of the negative control) [19]. The final OD of each tested strain was calculated as the OD average of the strain minus ODc. According to the calculated values, isolates were divided into four groups: no (N) biofilm producer (OD ≤ ODc), weak (W) biofilm producer (ODc < OD ≤ 2 × ODc), moderate (M) biofilm producer (2 × ODc < OD ≤ 4 × ODc), and strong (S) biofilm producer (4 × ODc < OD).

A swarming motility assay was performed with freshly grown cultures transferred with a sterile toothpick onto the surface of modified LB agar (tryptone—10 g/L; NaCl—5 g/L; yeast extract—5 g/L) with 0.4% agar [22]. The modified LB plates were prepared on the same day of the experiment and after inoculation, were incubated for 48 h at 37 °C. If the zone around the point of inoculation was greater than 10 mm, the isolate was considered positive for swimming motility.

## 3. Results

### 3.1. Pulsed Field Gel Electrophoresis (PFGE)

**Figure 2:**
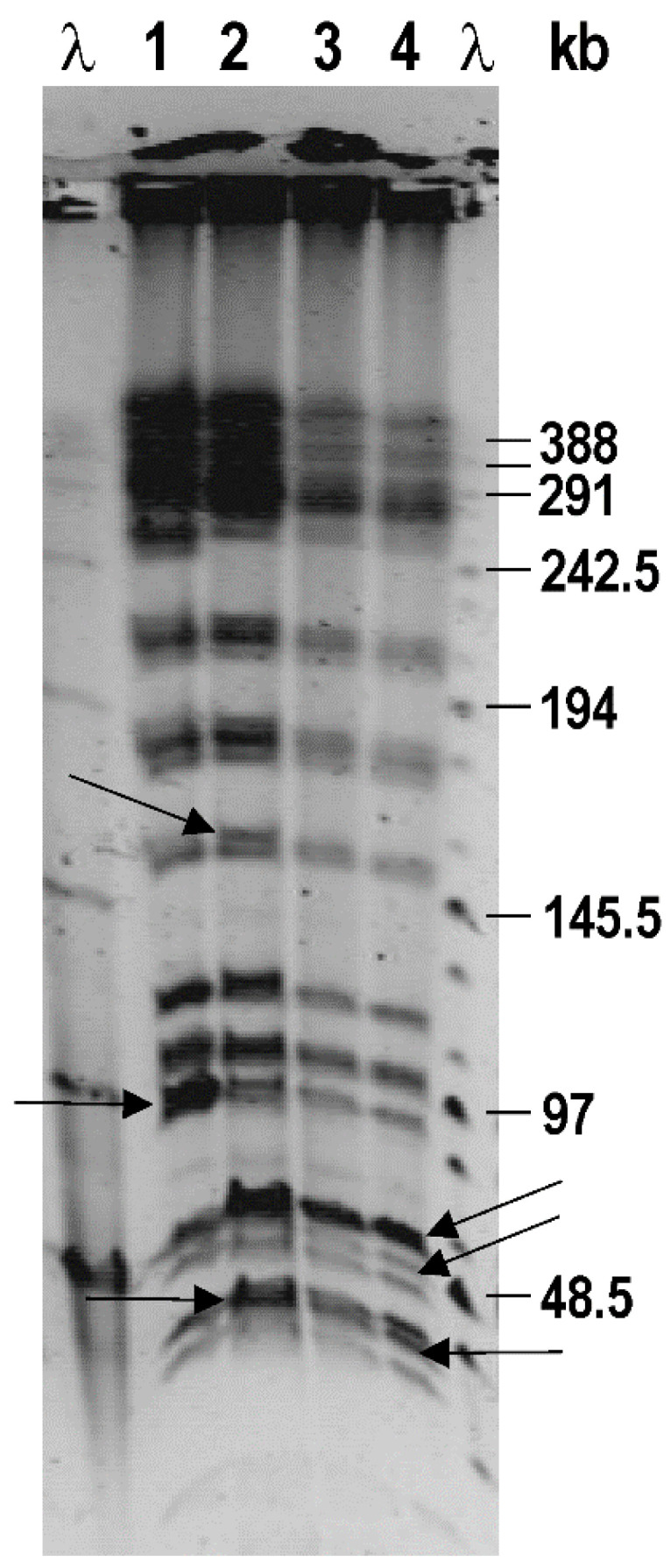
DNA patterns of *E. coli* isolates obtained by PFGE (*Xba*I macrorestriction fingerprint). λ-ladder (λ-concatemers; New England Biolabs), 1—strain 5381a; 2—strain 5381b; 3—strain 5681; 4—strain 5848. The differences between the strains are indicated by the arrows.

The DNA pattern of *E. coli* isolates obtained by the PFGE *Xba*I fingerprint is shown in Figure 2.

From the *Xba*I macro restriction pattern, it can be seen that the isolates show a high similarity in terms of restriction profile, which indicates a common origin. Differences between isolates refer to specific DNA fragments probably deriving from different extrachromosomal elements, that also show a high level of similarity. According to the *Xba*I pattern, it can be concluded that the analysed isolates 5681 and 5848 are highly similar and differ from 5381a and 5381b.

### 3.2. Sequence Analyses

#### 3.2.1. Species Identification and the Main Characteristics of Analysed Genomes

The species identification of collected isolates was confirmed using the sequences of the assembled genomes and publicly available databases. All four isolates were identified as *E. coli* (100%) serotype O25H4 using the Species ID tool from the ‘Public databases for molecular typing and microbial genome diversity (PubMLST)’ and the ‘16S rRNA based species identification Species Finder 2.0’ (https://cge.food.dtu.dk/services/SpeciesFinder/, accessed on 10 March 2024) [23,24]. Genome statistics are presented in Table 2.

#### 3.2.2. Pan-Genome Analyses

**Figure 3:**
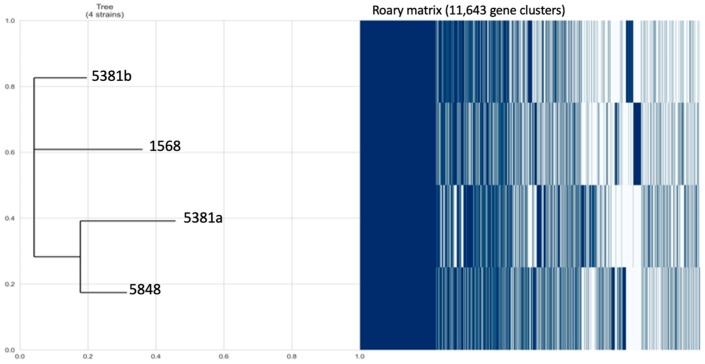
Visualisation of the pan-genome constructed using Roary based on the core and accessory genes showing phylogenetic relatedness of the isolates by blue (present) and white (absent) fragments.

Pan-genome analyses revealed a total of 11,643 gene clusters, which were classified into the following: 1. Hard core genes (i.e., that were present in >99% of the isolates, *n* = 5617); 2. Soft core genes that were present in 95–99% of isolates (*n* = 6026); 3. Accessory genes, which were further subdivided into shell genes, present in 15–95% of isolates (*n* = 0) and cloud genes, present in less than 15% (*n* = 0) (Supplementary Figure S1). The core genome of the four *E. coli* isolates confirmed that the analysed genomes were phylogenetically related, however, the isolates 5381a and 5848 were grouped together as they shared a high number of common genes (Figure 3).

#### 3.2.3. MLST Analyses of Sequenced Isolates

The four isolates belonged to an unknown sequence type (ST), that was related to *E. coli* ST131, a pandemic clone that is evolving rapidly with increasing levels of antimicrobial resistance [25], with five out of seven alleles being identical for isolates 5381a, 5681 and 5848, and six out of seven for isolate 5381b. Average nucleotide identity is presented in Supplementary Figure S2, while MLST analyses are presented in Table 3.

The comparison with the reference strain for *E. coli* ST131 revealed that the four strains isolated in this study presented 738 single nucleotide polymorphisms (SNPs), four of which were polymorphic. By comparing the SNP data with the clinically relevant genes, we found that 57 genes were mutated in at least one isolate (Table 4).

**Figure 4:**
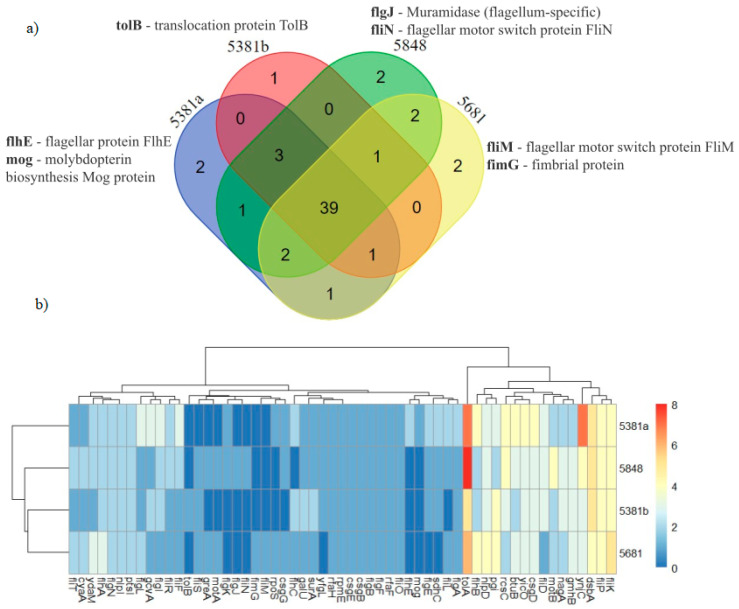
Single nucleotide polymorphisms (SNPs) occurring in clinically relevant genes: (**a**) the number of exclusive and shared mutated genes among the four strains; (**b**) the number of mutations within each gene, represented as a heatmap.

Most of the mutated genes were common to all four isolates, with a few mutations exclusive to individual isolates, and the number of mutations was not homogeneous among all the mutated genes (Figure 4).

All four genomes presented a well-defined contig of approximately 116 kb (in strain 5381a, this was 112 kb) that displayed the highest total score with the plasmid pMB2910_1 of 208,091 bp (GenBank Accession No. NZ_CP103740) (along with 100% query coverage and 99.7% ID). In strain 5381a, alignment using Mauve [26] revealed that the plasmid presented a well-defined region of approximately 5 kb that underwent inversion (Supplementary Figure S3).

### 3.3. Antibiotic Resistance Profiles of the Isolates

#### 3.3.1. Analyses of Antibiotic Resistance Genes Using ResFinder

Genes that determine the molecular basis of antibiotic resistance were detected by ResFinder [27]. The acquired antibiotic resistance genes according to the ResFinder database for all tested isolates are presented in Table 5.

#### 3.3.2. PCA of Antibiotic Resistance Molecular Determinants Detected by ResFinder Tool

**Figure 5:**
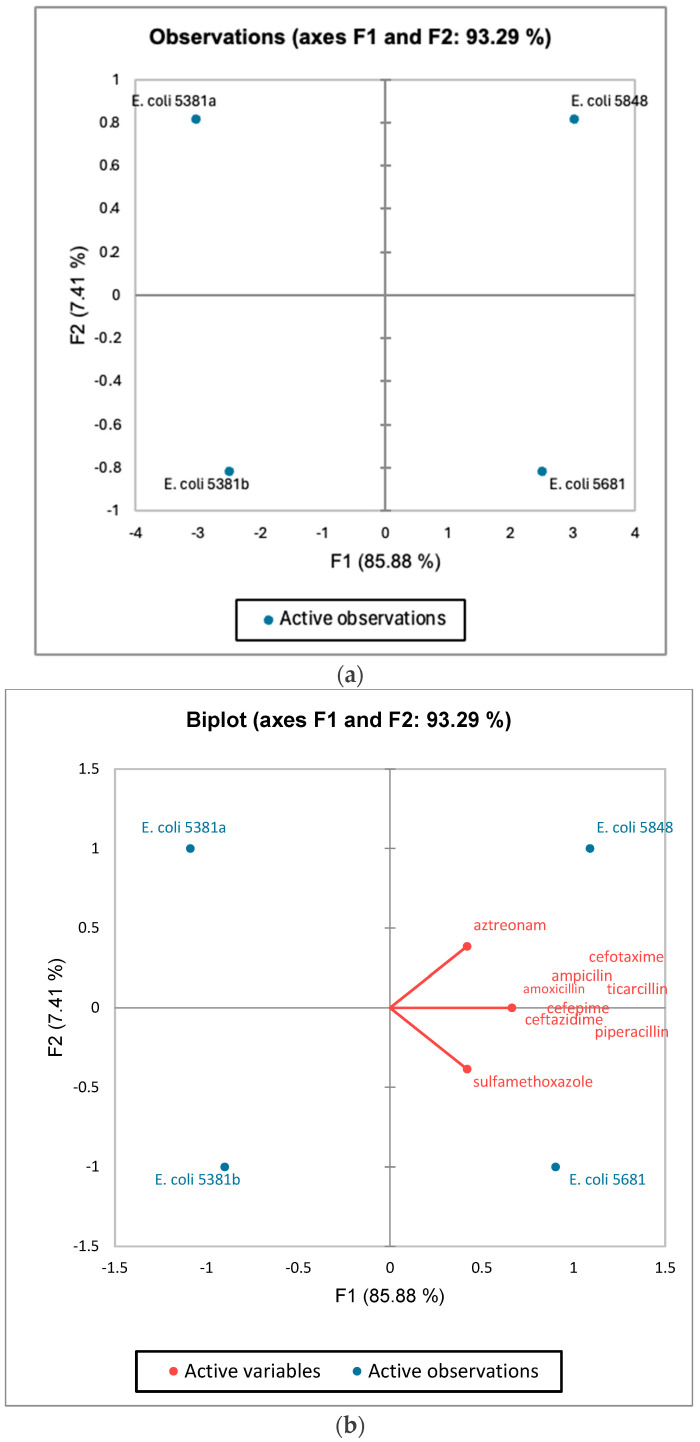
The individual factor map (**a**) and variables factor map (**b**) of PCA on different genetic determinants of antibiotic resistance, detected in genomes by the ResFinder database.

The PCA biplot based on the presence AMR genes detected by the ResFinder tool is presented in Figure 5. Dimension 1 (F1) describes 52.24% of the variability between the isolates *E. coli* 5381a and *E. coli* 5381b (characterised by a negative coordinate on the axis) and the isolate *E. coli* 5848 (characterised by a positive coordinate on the axis). Dimension 2 (F2) describes 7.41% of the variability between the isolates *E. coli* 5681 and *E. coli* 5848 and the other isolates *E. coli* 5381a and *E. coli* 5381b (Figure 5). According to Figure 5, using the ResFinder database, the isolates *E. coli* 5681 and *E. coli* 5848 had the highest number of genes coding for molecular determinants of antibiotic resistance [27].

#### 3.3.3. Antimicrobial Susceptibility Testing

All isolates were tested against eight different antibiotics belonging to different classes/subclasses of antibiotics—cephalosporins (ceftriaxone), carbapenems (meropenem), fluoroquinolones (ciprofloxacin), aminoglycosides (amikacin, gentamicin), and tetracyclines (doxycycline, minocycline, tetracycline). All isolates were resistant to ceftriaxone, ciprofloxacin, doxycycline, minocycline, and tetracycline (Table 6) and can be classified as multidrug-resistant (MDR) isolates as they are “resistant to three or more antimicrobial classes” [28].

### 3.4. Biofilm Formation and Swimming Motility Assay

Under the tested conditions, the isolates (5381a, 5381b and 5681) were classified as moderate (M) biofilm producers, while the latest obtained isolate (5848) was classified as a strong (S) biofilm producer (Table 7). Swimming motility was successively higher in relation to the time of isolation (Table 7).

### 3.5. Comparison of Detected Virulence Factors in Sequenced Genomes

Sequence data analysis by the VFDB online software (http://www.mgc.ac.cn/cgi-bin/VFs/v5/main.cgi, accessed on 10 June 2024) revealed genes coding for the following: adherence factors (*E. coli* common pilus-ECP, haemorrhagic *E. coli* pilus-HCP, P fimbriae, type I fimbriae), autotransporters (lateral flagella, contact-dependent inhibition CDI system), invasion, iron uptake (aerobactin siderophore, haem uptake), secretion system (SCI-I T6SS), and toxin(s) (enterotoxin SenB/TieB-present only in strain 5848, haemolysin/cytolysin A-present in all analysed genomes).

**Figure 6:**
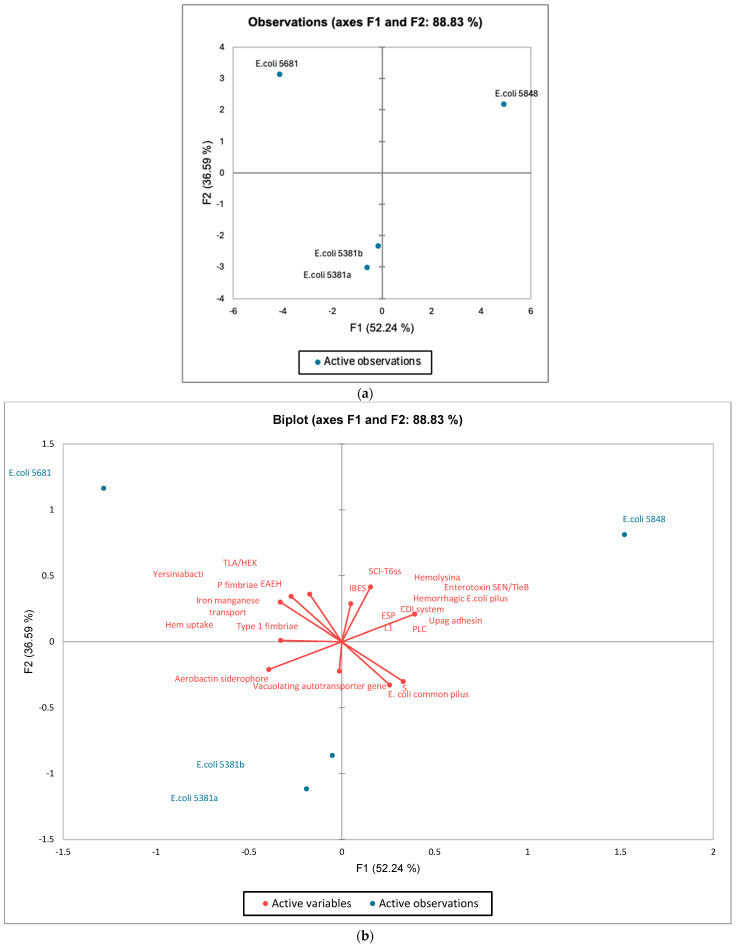
The individual factor map (**a**) and variables factor map (**b**) of PCA on different virulence factors, detected in genomes by VFDB.

The PCA plot, based on the presence of the virulence factor determinants detected by VFDB, is presented in Figure 6. Dimension 1 (F1) describes 52.24% of the variability between the isolates *E. coli* 5381a and *E. coli* 5381b (characterised by a negative coordinate on the axis) and the isolate *E. coli* 5848 (characterised by a positive coordinate on the axis). Isolates *E. coli* 5381a and *E. coli* 5381b have a strong correlation as they are grouped together (Figure 6). Dimension 2 (F2) describes 36.59% of the variability between the isolates *E. coli* 5681 and *E. coli* 5848 and the other isolates *E. coli* 5381a and *E. coli* 5381b (Figure 6). According to Figure 6, isolates *E. coli* 5681 and *E. coli* 5848 had the highest number of genes coding for virulence factors recognised by the VFDB database.

## 4. Discussion

In this longitudinal study, we present comparative analyses of four *E. coli* genomes from isolates recovered over a three week period from the tracheal aspirates sampled from a tracheotomised paediatric patient on chronic respiratory support. Clinical indicators suggest that the lower respiratory tract was colonised by *E. coli*. Although colonisation is typically considered the first step in microbial infection, colonisation and infection are undoubtedly two different processes. Colonisation is the presence of bacteria on the surface, or within the human body (e.g., the skin, intestines, airways, etc.), without causing symptoms of disease. In contrast, infection is the invasion of body tissues and the consequent development of disease [29]. *E. coli* is a common commensal bacterium within the intestine and is among the first colonising bacteria in the gut after birth [30]. However, in immunocompromised patients or in healthy individuals whose physical, anatomical, and physiological barriers have been compromised, *E. coli* can cause different intestinal and extraintestinal infections [30], as mentioned previously. In addition, the high genome plasticity of *E. coli* enables this bacterium to gain and lose genes through genetic changes, which means that it has the capacity to evolve from a commensal strain to a pathogenic strain. The colonisation of the upper respiratory tract of neonates by *E. coli* is possible due to contact with the maternal perineum at birth, but the incidence declines after the first year of age. In this study, we obtained *E. coli* isolates from the tracheal aspirates of a tracheotomised 3-year-old child, and analyses revealed the drug resistance and virulence profiles of the tested isolates. The colonisation of the lower respiratory tract, particularly by MDR pathogens as we see here, is clinically relevant, as these colonisations can act as a reservoir for future infection [31,32]. Understanding the genetic background of the colonising isolates and how they change over time is, therefore, key to understanding the transition from colonisation to infection.

Although the time period of the bacterial collection was short, this study evaluated *E. coli* strain adaptation in a hospitalised child within the nosocomial environment. According to PFGE and Pan-Genome analyses, all four isolates had a high percentage of similarity and shared a high number of common chromosomal and plasmid genes, which were expected given that all isolates were collected within a short period of time.

On the other hand, MLST analyses revealed that all four strains belonged to an unknown ST, but with a high similarity to *E. coli* ST131, which is recognised as a pandemic multidrug resistant (MDR) strain that has had unprecedented global expansion in the last decade and is now the predominant resistant *E. coli* in both the adult and paediatric populations [33]. *E. coli* ST131 is the dominant multi-drug resistant (MDR) clone among extraintestinal pathogenic *E. coli* (ExPEC) isolates today, so it presents a major public health risk and is classified as a critical priority pathogen by the World Health Organization (WHO) [34]. In addition, most of the *E. coli* ST131 isolates carry mobile genetic elements encoding for the synthesis of the siderophores aerobactin (*iuc*) and yersiniabactin (*ybt*), two important factors promoting extraintestinal colonisation [35,36]. Both of these genes (*iuc* and *ybt*) were detected in all of the *E. coli* genomes analysed in this study using VFDB (Supplementary Data) [20], without detectable mutations when compared with the wild type strain for ST131 (Table 4).

Although recovered in a short time frame, all four *E. coli* isolates in this study exhibited a MDR phenotype, as according to MIC values, all were resistant to ceftriaxone, ciprofloxacin, doxycycline, minocycline, and tetracycline. In addition, PCA revealed that isolates 5681 and 5848, which were recovered later than 5381a and 5381b (two weeks and three weeks, respectively) possessed a more complex antibiotic resistance profile (Figure 5b), which is extremely concerning considering the short time period between the isolation of the strains. Adaptation to one environment, in this case adaption within the same patient, can generate phenotypic and genotypic changes which contribute to the future ability of an organism to survive environmental conditions [37]. We speculate that this is what arguably had happened with the isolates analysed in this study, since there is a difference in antibiotic resistance profiles among isolates (each subsequent isolate had an increased level of AMR genes), and the latest isolate (5848) had the ability to form strong biofilms (Table 7). Biofilm formation can increase survival rates in vivo upon exposure to stresses, including the host’s immune system or antibiotic therapy. Biofilm formation presents a successful survival strategy and is a central microbial characteristic for evolution and adaption [38].

PFGE analysis showed great similarity, and thus a common origin of the isolated strains. The newly isolated strains differed from *E. coli* ST131 for 738 SNPs. In addition to common mutations, mutations unique to certain strains were also observed. A small plasmid inversion was also detected, which indicated a great dynamism in the plasticity of the genome that contributes to the adaptability of the isolates [39]. These mutations could result in phenotypic changes, such as enhanced biofilm formation, antibiotic resistance, and motility. Long-term studies of infection, such as a 20-year longitudinal study of *Pseudomonas aeruginosa* in a patient with cystic fibrosis [40], reveal various mutations and genetic rearrangements between isolates collected over the course of a chronic infection. Here, we find genomic variations between isolates collected over only three weeks in a patient with respiratory colonisation.

## 5. Conclusions

It can be concluded that the results of our study confirmed the MDR phenotype and virulence potential of the analysed *E. coli* isolates collected longitudinally from a paediatric patient, as well as chromosomal and plasmid changes. In addition to the acquisition of new genes, it is possible that point mutations contribute to changes in the expression of genes associated with virulence factors, motility, biofilm formation, and antibiotic resistance. The tested isolates belong to an unknown strain, however they are related to the *E. coli* ST131, a pandemic MDR strain which is also a dominant extraintestinal pathogenic strain. Through longitudinal studies of MDR bacteria colonising the lower respiratory tract, such as the *E. coli* isolates studied here, we can better understand the genomic changes that occur during colonisation prior to infection.

## Figures and Tables

**Table 1:** Labelling, date of sampling, and origin of *E. coli* isolates.

Labelling	Date of Sampling	Specimen	Isolate
1. 5381a	3 August 2020	Tracheal aspirate	*Escherichia coli*
2. 5381b	3 August 2020	Tracheal aspirate	*Escherichia coli*
3. 5681	17 August 2020	Tracheal aspirate	*Escherichia coli*
4. 5848	24 August 2020	Tracheal aspirate	*Escherichia coli*

**Table 2:** The main characteristics of the analysed genomes.

Genome	Size	%GC	Number of Contigs	N50	Number of Predicted Genes	CheckM %Compl.	CheckM %Cont.
5381a	5,357,118	50.78 ± 2.09	3	5.240.079	8535	91.58	1.45
5381b	5,454,155	50.68 ± 0.64	2	5.337.429	8176	93.71	0.54
5681	5,483,488	50.71 ± 1.89	5	3.064.587	8224	94.22	1.27
5848	5,451,275	50.69 ± 0.64	2	5.334.559	8685	89.71	1.61

**Table 3:** MLST analyses of tested genomes.

Genome	adk	fumC	gyrB	icd	mdh	purA	recA
5381a	53	40	47 *	13	36 **	28	29
5381b	53	40	47	13	36 **	28	29
5681	53	40	47 *	13	36 **	28	29
5848	53	40	47 *	13	36 **	28	29

* Has the same indel in 3 out of 4 isolates; ** Has the same indel in all four isolates.

**Table 4:** A list of the clinically relevant genes found to be mutated in at least one isolate.

Gene	Product	Gene	Product
yicO	putative permease	flgE	flagellar hook protein FlgE
rfaF	ADP-heptose—LPS-heptosyltransferase II	flgB	flagellar basal-body rod protein FlgB
nlpI	lipoprotein NlpI precursor	flgA	flagella basal body P-ring formation protein FlgA
gcvA	putative transcriptional regulator of glycine cleavage system	flgN	flagella synthesis protein FlgN
nlpD	lipoprotein NlpD	csgB	curlin minor subunit
rpoS	RNA polymerase sigma factor RpoS	csgD	csgab operon transcriptional regulator
yfgL	putative dehydrogenase	csgE	curli production assembly/transport component
ptsI	PEP-protein phosphotransferase system enzyme I	csgG	curli production assembly/transport component
rcsC	two-component system sensor kinase	tolA	TolA protein
fliR	flagellar biosynthesis protein FliR	sdhC	succinate dehydrogenase cytochrome b556 large membrane subunit
fliP	flagellar biosynthetic protein FliP	nagA	N-acetylglucosamine-6-phosphate deacetylase
fliO	flagellar biosynthesis protein FliO	gmhB	D,D-heptose 1,7-bisphosphate phosphatase
fliL	flagellar protein FliL	surA	chaperone precursor (peptidyl-prolyl cis-trans isomerase)
fliK	flagellar hook-length control protein	mog	molybdopterin biosynthesis Mog protein
fliF	flagellar M-ring protein	Pgi	glucosephosphate isomerase
fliT	flagellar protein FliT	btuB	vitamin B12/cobalamin outer membrane transporter
fliD	flagellar capping protein	rpmE	50S ribosomal protein L31
flhC	flagellar transcriptional activator	dsbA	thiol:disulfide interchange protein
motB	chemotaxis protein MotB (motility protein B)	rfaH	transcriptional activator
flhB	flagellar biosynthesis protein FlhB	cyaA	adenylate cyclase
flhA	flagellar biosynthesis protein FlhA	fliS	flagellar protein FliS
flhE	flagellar protein FlhE	tolB	translocation protein TolB
ynjC	ABC transporter permease	flgJ	Muramidase (flagellum-specific)
ydaM	putative signaling protein	fliN	flagellar motor switch protein FliN
galU	UTP—glucose-1-phosphate uridylyltransferase	greA	transcription elongation factor GreA
flgL	flagellar hook-associated protein 3	motA	chemotaxis protein MotA (motility protein A)
flgK	flagellar hook-associated protein 1	fimG	fimbrial protein
flgI	flagellar P-ring protein	fliM	flagellar motor switch protein FliM
flgF	flagellar basal-body rod protein FlgF		

**Table 5:** Acquired antibiotic resistance genes according to the ResFinder database.

Antibiotic Class	Gene/Gene Location			
	5381a	5381b	5681	5848
Aminoglycoside	*aac(3)-IIa*/plasmid	*aac(3)-IIa*/plasmid *aadA5*/plasmid	*aac(3)-IIa*/plasmid *aadA5*/plasmid	*aac(3)-IIa*/plasmid *aadA5*/plasmid
Macrolide	*mph(A)*/plasmid	*mph(A)*/plasmid	*mph(A)*/plasmid	*mph(A)*/plasmid
Quinolone	*aac(6′)-Ib-cr*/plasmid	*aac(6′)-Ib-cr*/plasmid	*aac(6′)-Ib-cr*/plasmid	*aac(6′)-Ib-cr*/plasmid
Peroxide	*sitABCD*/chromosome	*sitABCD*/chromosome	*sitABCD*/chromosome	*sitABCD*/chromosome
Beta-lactam	*bla*_CTX-M-15_/plasmid *bla*_OXA-1_/plasmid	*bla*_CTX-M-15_/plasmid *bla*_OXA-1_/plasmid	*bla*_CTX-M-15_/plasmid *bla*_OXA-1_/plasmid	*bla*_CTX-M-15_/plasmid *bla*_OXA-1_/plasmid
Amphenicol	*catB3*/plasmid	*catB3*/plasmid	*catB3*/plasmid	*catB3*/plasmid
Folate pathway antagonist	/	*dfrA17*/plasmid *sul1*/plasmid	*dfrA17*/plasmid *sul1*/plasmid	*dfrA17*/plasmid *sul1*/plasmid
Aminocyclitol	/	*aadA5*/plasmid	*aadA5*/plasmid	*aadA5*/plasmid
Quaternary ammonium compound	/	*qacE*/plasmid	*qacE*/plasmid	*qacE*/plasmid

**Table 6:** MIC values and resistance profiles of tested isolates.

Antibiotic	5381a	5381b	5681	5848
Ceftriaxone *	MIC > 4 μg/mL (R)	MIC > 4 μg/mL (R)	MIC > 4 μg/mL (R)	MIC > 4 μg/mL (R)
Meropenem *	MIC < 1 μg/mL (S)	MIC < 1 μg/mL (S)	MIC < 1 μg/mL (S)	MIC < 1 μg/mL (S)
Ciprofloxacin *	MIC > 1 μg/mL (R)	MIC > 1 μg/mL (R)	MIC > 1 μg/mL (R)	MIC > 1 μg/mL (R)
Amikacin *	MIC = 8 μg/mL (S)	MIC = 8 μg/mL (S)	MIC = 8 μg/mL (S)	MIC = 8 μg/mL (S)
Gentamicin *	MIC < 1 μg/mL (S)	MIC < 1 μg/mL (S)	MIC < 1 μg/mL (S)	MIC < 1 μg/mL (S)
Doxycycline **	MIC > 32 μg/mL (R)	MIC > 32 μg/mL (R)	MIC > 32 μg/mL (R)	MIC > 32 μg/mL (R)
Minocycline **	MIC > 32 μg/mL (R)	MIC > 32 μg/mL (R)	MIC > 32 μg/mL (R)	MIC > 32 μg/mL (R)
Tetracycline **	MIC > 32 μg/mL (R)	MIC > 32 μg/mL (R)	MIC > 32 μg/mL (R)	MIC > 32 μg/mL (R)

Abbreviations: MIC (minimal inhibitory concentration); R—resistant; S—sensitive; *—breakpoints from EUCAST; **—breakpoints from CLSI.

**Table 7:** The biofilm formation and swimming motility of tested isolates.

Isolate	Biofilm Formation	Swimming Motility (mm)
5381a	Moderate (M)	11
5381b	Moderate (M)	12
5681	Moderate (M)	15
5848	Strong (S)	30

## Data Availability

The original contributions presented in the study are included in the article/supplementary material, further inquiries can be directed to the corresponding author.

## Supplementary Materials

The following supporting information can be downloaded at: https://www.mdpi.com/article/10.3390/microorganisms12101990/s1, Figure S1: Pan-genome analyses of assembled genomes and number of core and shell genes. Figure S2: Average nucleotide identity between the genome sequences of *E. coli* isolates. Figure S3: Alignment of the plasmid homologous to pMB2910_1 in the four strains, in order from top: 5381a, 5381b, 5681, and 5848.

## References

[1] Yayan J., Ghebremedhin B., Rasche K. (2015). No Development of Imipenem Resistance in Pneumonia Caused by *Escherichia coli*. Medicine.

[2] Schneer S., Khoury J., Adir Y., Stein N., Shaked Mishan P., Ken-Dror S., Weber G., Meler R., Khateeb A., Shteinberg M. (2020). Clinical characteristics and outcomes of patients with *Escherichia coli* in airway samples. Clin. Respir. J..

[3] John T.M., Deshpande A., Brizendine K., Yu P.C., Rothberg M.B. (2021). Epidemiology and Outcomes of Community-Acquired *Escherichia coli* Pneumonia. Open Forum Infect. Dis..

[4] Li F., Zhu J., Zheng Y., Fang Y., Hu L., Xiong J. (2024). Comparison of bacteremic pneumonia caused by *Escherichia coli* and Klebsiella pneumoniae: A retrospective study. Saudi Med. J..

[5] Sherchan J.B., Humagain S. (2021). Antimicrobial Susceptibility Pattern of Gram-Negative Bacteria Causing Lower Respiratory Tract Infections in Kathmandu University Hospital. J. Nepal Health Res. Counc..

[6] Quarata F., Lelli D., Biancone D.M., Gherardi G., Incalzi R.A. (2023). A urinary tract infection caused by *Escherichia coli* mucoid phenotype progresses to a pneumonia and respiratory failure. Lancet.

[7] Jovcic B., Lepsanovic Z., Suljagic V., Rackov G., Begovic J., Topisirovic L., Kojic M. (2011). Emergence of NDM-1 metallo-Œ≤-lactamase in *Pseudomonas aeruginosa* clinical isolates from Serbia. Antimicrob. Agents Chemother..

[8] Barton B.M., Harding G.P., Zuccarelli A.J. (1995). A general method for detecting and sizing large plasmids. Anal. Biochem..

[9] Kolmogorov M., Yuan J., Lin Y., Pevzner P.A. (2019). Assembly of long, error-prone reads using repeat graphs. Nat. Biotechnol..

[10] Gurevich A., Saveliev V., Vyahhi N., Tesler G. (2013). QUAST: Quality assessment tool for genome assemblies. Bioinformatics.

[11] Vaser R., Sović I., Nagarajan N., Šikić M. (2017). Fast and accurate de novo genome assembly from long uncorrected reads. Genome Res..

[12] Pritchard L., Glover R.H., Humphris S., Elphinstone J.G., Toth I.K. (2016). Genomics and taxonomy in diagnostics for food security: Soft-rotting enterobacterial plant pathogens. Anal. Methods.

[13] Esposito A., Tamburini S., Triboli L., Ambrosino L., Chiusano M.L., Jousson O. (2019). Insights into the genome structure of four acetogenic bacteria with specific reference to the Wood-Ljungdahl pathway. Microbiologyopen.

[14] Decano A.G., Ludden C., Feltwell T., Judge K., Parkhill J., Downing T. (2019). Complete Assembly of *Escherichia coli* Sequence Type 131 Genomes Using Long Reads Demonstrates Antibiotic Resistance Gene Variation within Diverse Plasmid and Chromosomal Contexts. mSphere.

[15] Ludden C., Decano A.G., Jamrozy D., Pickard D., Morris D., Parkhill J., Peacock S.J., Cormican M., Downing T. (2020). Genomic surveillance of *Escherichia coli* ST131 identifies local expansion and serial replacement of subclones. Microb. Genom..

[16] Niba E.T., Naka Y., Nagase M., Mori H., Kitakawa M. (2007). A genome-wide approach to identify the genes involved in biofilm formation in *E. coli*. DNA Res..

[17] Jovčić B., Novović K., Filipić B., Velhner M., Todorović D., Matović K., Rašić Z., Nikolić S., Kiškarolj F., Kojić M. (2020). Genomic Characteristics of Colistin-Resistant *Salmonella enterica* subsp. *enterica* Serovar Infantis from Poultry Farms in the Republic of Serbia. Antibiotics.

[18] Page A.J., Cummins C.A., Hunt M., Wong V.K., Reuter S., Holden M.T., Fookes M., Falush D., Keane J.A., Parkhill J. (2015). Roary: Rapid large-scale prokaryote pan genome analysis. Bioinformatics.

[19] Bortolaia V., Kaas R.S., Ruppe E., Roberts M.C., Schwarz S., Cattoir V., Philippon A., Allesoe R.L., Rebelo A.R., Florensa A.R. (2020). ResFinder 4.0 for predictions of phenotypes from genotypes. J. Antimicrob. Chemother..

[20] Liu B., Zheng D., Zhou S., Chen L., Yang J. (2022). VFDB 2022: A general classification scheme for bacterial virulence factors. Nucleic Acids Res..

[21] Stepanović S., Vuković D., Hola V., Di Bonaventura G., Djukić S., Ćirković I., Ruzicka F. (2007). Quantification of biofilm in microtiter plates: Overview of testing conditions and practical recommendations for assessment of biofilm production by staphylococci. APMIS.

[22] Swiecicki J.M., Sliusarenko O., Weibel D.B. (2013). From swimming to swarming: *Escherichia coli* cell motility in two-dimensions. Integr. Biol..

[23] Larsen M.V., Cosentino S., Lukjancenko O., Saputra D., Rasmussen S., Hasman H., Sicheritz-Pontén T., Aarestrup F.M., Ussery D.W., Lund O. (2014). Benchmarking of methods for genomic taxonomy. J. Clin. Microbiol..

[24] Jolley K.A., Bray J.E., Maiden M.C.J. (2018). Open-access bacterial population genomics: BIGSdb software, the PubMLST.org website and their applications. Wellcome Open Res..

[25] Pitout J.D., DeVinney R. (2017). *Escherichia coli* ST131: A multidrug-resistant clone primed for global domination. F1000Research.

[26] Darling A.C., Mau B., Blattner F.R., Perna N.T. (2004). Mauve: Multiple alignment of conserved genomic sequence with rearrangements. Genome Res..

[27] Florensa A.F., Kaas R.S., Clausen P.T.L.C., Aytan-Aktug D., Aarestrup F.M. (2022). ResFinder—An open online resource for identification of antimicrobial resistance genes in next-generation sequencing data and prediction of phenotypes from genotypes. Microb. Genom..

[28] Magiorakos A.P., Srinivasan A., Carey R.B., Carmeli Y., Falagas M.E., Giske C.G., Harbarth S., Hindler J.F., Kahlmeter G., Olsson-Liljequist B. (2012). Multidrug-resistant, extensively drug-resistant and pandrug-resistant bacteria: An international expert proposal for interim standard definitions for acquired resistance. Clin. Microbiol. Infect..

[29] Dani A. (2014). Colonization and infection. Cent. Eur. J. Urol..

[30] Pokharel P., Dhakal S., Dozois C.M. (2023). The Diversity of *Escherichia coli* Pathotypes and Vaccination Strategies against This Versatile Bacterial Pathogen. Microorganisms.

[31] Siegel S.J., Weiser J.N. (2015). Mechanisms of Bacterial Colonization of the Respiratory Tract. Annu. Rev. Microbiol..

[32] Claassen-Weitz S., Lim K.Y.L., Mullally C., Zar H.J., Nicol M.P. (2021). The association between bacteria colonizing the upper respiratory tract and lower respiratory tract infection in young children: A systematic review and meta-analysis. Clin. Microbiol. Infect..

[33] Whitmer G.R., Moorthy G., Arshad M. (2019). The pandemic *Escherichia coli* sequence type 131 strain is acquired even in the absence of antibiotic exposure. PLoS Pathog..

[34] Biggel M., Moons P., Nguyen M.N., Goossens H., Van Puyvelde S. (2022). Convergence of virulence and antimicrobial resistance in increasingly prevalent *Escherichia coli* ST131 papGII+ sublineages. Commun. Biol..

[35] Gao Q., Wang X., Xu H., Xu Y., Ling J., Zhang D., Gao S., Liu X. (2012). Roles of iron acquisition systems in virulence of extraintestinal pathogenic *Escherichia coli*: Salmochelin and aerobactin contribute more to virulence than heme in a chicken infection model. BMC Microbiol..

[36] Galardini M., Clermont O., Baron A., Busby B., Dion S., Schubert S., Beltrao P., Denamur E. (2020). Major role of iron uptake systems in the intrinsic extra-intestinal virulence of the genus Escherichia revealed by a genome-wide association study. PLoS Genet..

[37] Nucci A., Rocha E.P.C., Rendueles O. (2023). Latent evolution of biofilm formation depends on life-history and genetic background. NPJ Biofilms Microbiomes.

[38] Parrilli E., Tutino M.L., Marino G. (2022). Biofilm as an adaptation strategy to extreme conditions. Rend. Lincei. Sci. Fis. Nat..

[39] Horton J.S., Taylor T.B. (2023). Mutation bias and adaptation in bacteria. Microbiology.

[40] Wardell S.J.T., Gauthier J., Martin L.W., Potvin M., Brockway B., Levesque R.C., Lamont I.L. (2021). Genome evolution drives transcriptomic and phenotypic adaptation in *Pseudomonas aeruginosa* during 20 years of infection. Microb. Genom..

