# Supplementary material for: A Longitudinal Study of Escherichia coli Clinical Isolates from the Tracheal Aspirates of a Paediatric Patient—Strain Type Similar to Pandemic ST131
Source: Microorganisms. 2024 Sep 30;12(10):1990. doi: 10.3390/microorganisms12101990 (PMC11509341; doi:10.3390/microorganisms12101990)
Supplement: Supplementary file 1 [file microorganisms-12-01990-s001.zip › microorganisms-3185249-supplementary.pdf]

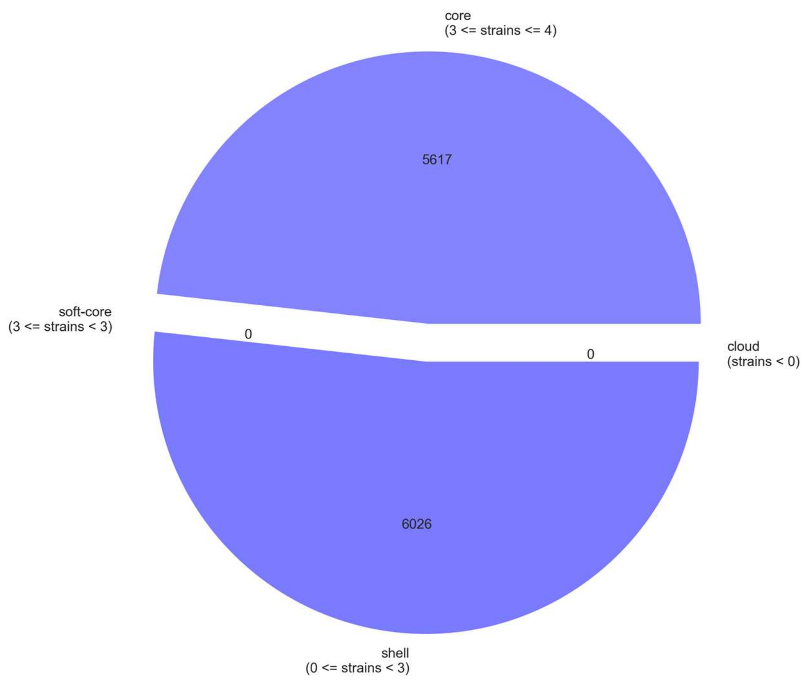

Supplementary Figure S1: Pan-genome analyses of assembled genomes and number of core and shell genes.

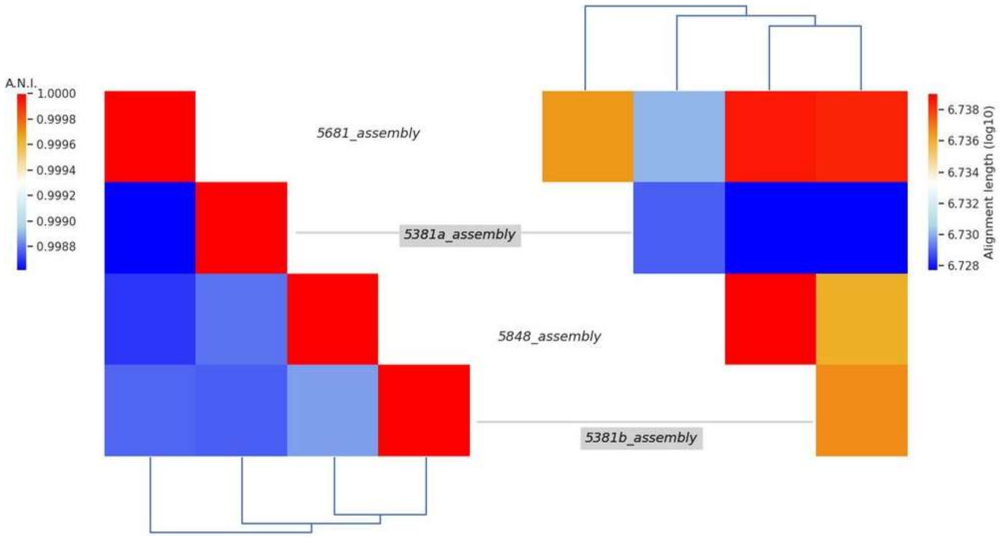

Supplementary Figure S2. Average nucleotide identity between genome sequences of *E. coli* isolates.

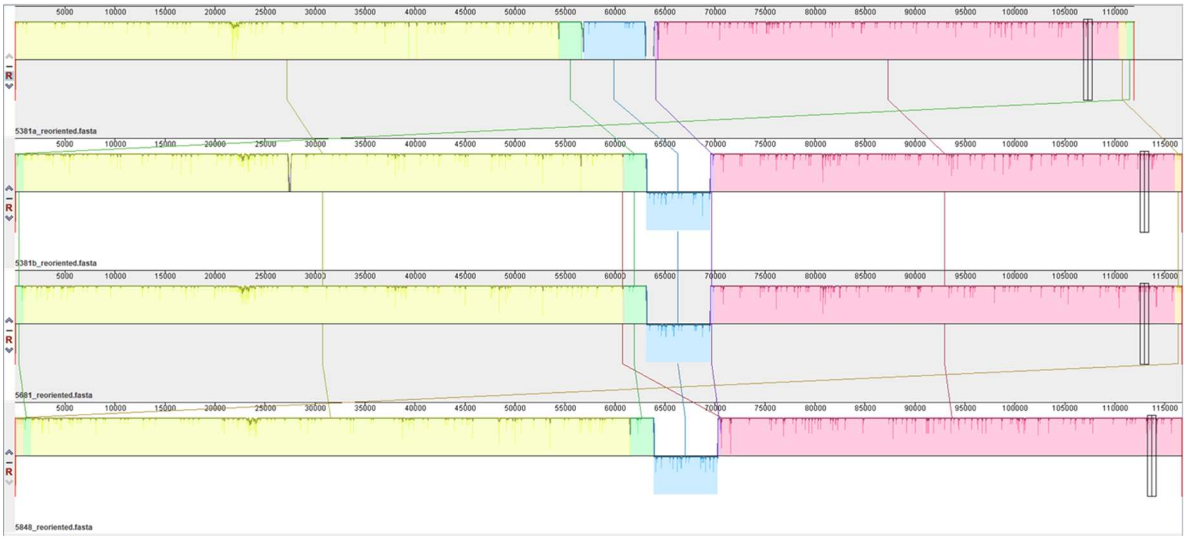

Supplementary Figure S3. Alignment of the plasmid homologous to pMB2910_1 in the four strains, in order from top: 5381a, 5381b, 5681 and 5848.
